# Solution Synthesis of Co-Ni-W-Based ODS Alloy Powder

**DOI:** 10.3390/ma12081231

**Published:** 2019-04-15

**Authors:** Lin Zhang, Ye Liu, Xiaowei Chen, Yan Chen, Shengxi Wang, Mingli Qin, Xuanhui Qu

**Affiliations:** 1 Beijing Advanced Innovation Center for Materials Genome Engineering, Institute for Advanced Materials and Technology, University of Science and Technology Beijing, Beijing 100083, China; s20161331@xs.ustb.edu.cn (X.C.); b20180575@xs.ustb.edu.cn (Y.C.); s20161386@xs.ustb.edu.cn (S.W.); qinml@mater.ustb.edu.cn (M.Q.); 2 School of Materials Science and Engineering, Xiangtan University, Xiangtan 411105, China; liuye@xtu.edu.cn

**Keywords:** Co-Ni-W-based powder, low-temperature combustion synthesis, oxide dispersion strengthening, particle size

## Abstract

Low-temperature combustion synthesis was utilized to prepare Co-Ni-W-based oxide dispersion strengthened (ODS) alloy powder. The influence of the U/Co and C/Co ratios on the morphology and specific surface area of the combusted powder was investigated. Particle size, phase constituents, and element distribution of the resulting Co-Ni-W-based ODS alloy powder were characterized. The results indicate that insufficient urea induced no autocombustion reaction, while excess urea and glucose inhibited the combustion reaction. The optimized contents of urea and glucose were around U/Co = 1.2 and C/Co = 1.5, and the specific surface area of the powder reached 43.5 m^2^/g. The lamellar Co-Ni-W-based ODS alloy powder with particle sizes of 1–21 μm was the soft agglomeration of a high population of nanosized (65 nm) particles. These nanoparticles grew from 65 to 260 nm in the reduction temperature range of 700–900 °C. Homogeneous distribution of Co, Ni, W, and Y in the Co-Ni-W-based ODS alloy powder was achieved.

## 1. Introduction

Co-based oxide dispersion strengthened (ODS) alloys exhibit superior high-temperature mechanical properties, which is expected to fulfill the strict requirement in some hot-section components for aerospace and power-generation applications [1,2,3,4]. Co-Ni-W-based ODS alloy was designed by the combination of γ’ [Co_3_(Al,W)] precipitates and oxide dispersion. W and Al acted as γ’ forming elements, while Ni was used to extend the solubility limit of Al and W in the cobalt matrix [5,6,7].

Mechanical alloying (MA) is the main technique used to fabricate Co-based ODS alloy powder [1,2,3]. Owing to the extremely low solubility of Al and W in cobalt matrix at room temperature, mechanical alloying induced the formation of supersaturated solid solution but not the fully alloyed powder, resulting in the formation of a low volume fraction of γ’ precipitates [4,5,6,7]. Moreover, the problems of energy and time consumption, as well as the high tendency of aggregation, were experienced during the MA process [8]. Therefore, the synthesis of Co-based ODS alloy powder with uniform dispersion of ultrafine oxides by a simple and easy route has received great attention. This is of great importance for the successful application of Co-based ODS alloys. 

Low-temperature combustion synthesis (LCS) has paved a new way for the production of ODS alloy powder, and the advantages of this technique can be seen from the following aspects [9,10,11,12]: Firstly, LCS is a kind of energy- and time-saving, simple, and cheap process. Secondly, an aqueous combustion reaction facilitates the mixture of raw materials at a molecular level, and the evolution of a large volume of gases during the combustion process is beneficial for improving the dispersibility of oxide dispersoids. Thirdly, nanosized ODS alloy powder exhibits high purity, high sinterability, high specific surface area, and well-defined chemical compositions.

The objective of this study is to investigate the potential of the LCS method for introducing nanosized oxide particles in a cobalt matrix. The key processing parameters were discussed, and the main focus was on the characterization of the combusted powder and the reduced powder.

## 2. Experimental Procedures

Analytical-grade cobalt nitrate (Co(NO_3_)_2_·6H_2_O), nickel nitrate (Ni(NO_3_)_2_6H_2_O), ammonium tungstate (NH_4_)_2_WO_4_, and yttrium nitrate (Y(NO_3_)_3_·6H_2_O) were the sources of Co, Ni, W, and Y, respectively. Urea (CH_4_N_2_O) was used as fuel for combustion, and glucose (C_6_H_12_O_6_·H_2_O) acted as dispersant [13]. The composition of the ODS alloys was designed to be Co-30Ni-20W-1.5Y_2_O_3_ (wt. %). Two categories of alloys were designed. In the first category of alloys, the C/Co ratio was constant at 1.5, and the U/Co ratio varied in the range of 0–3.0. In the second category of alloys, the U/Co ratio was constant at 1.2, and the C/Co ratios varied in the range of 0–3.0. Firstly, the starting solution was prepared by dissolving the above constituents into 200 mL distilled water or alcohol. Then, this solution was heated in air on an electrical furnace whose peak temperature was 300 °C. This mixture solution was evaporated at around 95 °C, and a clear brown-colored colloidal substance was gradually formed. The colloidal substance swelled and became foamy, and was then ignited. After combustion for several minutes, the mixture of Co_2_O_3_, NiO, WO_3_, Y_2_O_3_, and C were obtained through the following redox reactions:

9 Co(NO_3_)_2_ + 14 NH_2_CONH_2_ → 3 Co_3_O_4_ (s) + 22 H_2_O (g) + 14 CO_2_(g) + 23 N_2_ (g) (1)

9 Ni(NO_3_)_2_ + NH_2_CONH_2_ → 9 NiO + 14 N_2_ + 20 CO_2_ + 25 H_2_O (2)

2 Y(NO_3_)_3_ + 5 NH_2_CONH_2_→Y_2_O_3_ (s)+CO_2_ (g) + 2 H_2_O (g) + 2 N_2_ (g) (3)

5(NH_4_)_2_WO_4_ + 12HNO_3_ + 15NH_2_CONH_2_ + 20O_2_ →5WO_3_ (s) +15 CO_2_ (g) + 56 H_2_O (g) + 26 N_2_ (g) (4)

At the same time, the heat generated in the combustion reaction resulted in the glucose being dehydrated and carbonized to form carbon (Equation (5)), and the exothermic reaction between carbon and the oxygen took place in air (Equation (6)).

C_6_H_12_O_6_ (l) → 6 C (s) + 6 H_2_O (g) (5)

C (s) + O_2_ (g) → CO_2_ (g) (6)

Subsequently, the powder mixture was calcinated in a muffle furnace in order to remove the carbonic residues. Finally, the powder mixtures were reduced by high-purity hydrogen at the temperature range of 500–900 °C. Co_2_O_3_, NiO, and WO_3_ were converted to Co, Ni, and W, while the irreducible Y_2_O_3_ remained as the dispersoids.

The specific surface area (SSA) of the powder was measured using the BET method. The microstructure of the combusted powder and reduced products were observed on a JSM 7500F secondary electron microscopy (SEM, JEOL, Tokyo, Japan). Particle size and size distribution of the oxide were measured by image analyzing of the SEM images. Phase constituents were studied by Siemens D 5000 X-ray diffraction (XRD, Siemens, Munich, Germany) meter using Cu radiation, and the peaks were identified using ICDD database, 2009. Fourier transform infrared (FT-IR) spectra were recorded from 400 to 4000 cm^−1^ with a Nicolet NEXUS 670 Fourier Transform Infrared using the KBr pellet technique (Thermo Fisher Scientific Waltham, MA, USA).

## 3. Results and Discussion

### 3.1. Characterization of the Combusted Powder

**Figure 1:**
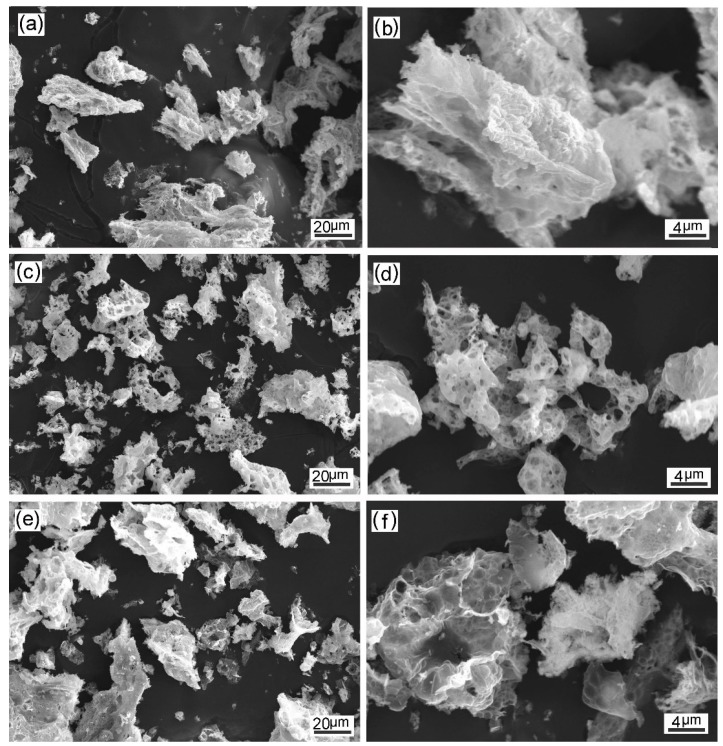
Secondary electron microscopy (SEM) images of the combusted powder made from the solution precursors with varied U/Co and C/Co molar ratios: (**a**,**b**) U/Co = 0.5, C/Co = 1.5; (**c**,**d**) U/Co = 1.2, C/Co = 1.5; (**e**,**f**) U/Co = 1.2; C/Co = 3.0.

The U/Co and C/Co ratios are the most important controlling parameters in the combustion reactions [14,15]. The content of urea is closely associated with the combustion temperature, while glucose is used to control the combustion temperature and to ameliorate the dispersibility of the powder. Figure 1 shows the SEM images of the combusted powder made from the precursors with varied compositions. The appearance of the combusted powder with C/Co = 1.5 and varied U/Co ratios is compared in Figure 1a,b and Figure 1c,d. In the case of low U/Co ratio (0.5), block particles with compact structure and a relatively large size of around 40–70 μm were formed, as displayed in Figure 1b. Figure 1c,d shows the morphology of the combusted powder under optimized condition (U/Co = 1.2, C/Co = 1.5). This powder exhibits the largest swelling, and small particles with an average size of about 16 μm were formed. The release of a large amount of gas promotes the fragmentation of the powder and the formation of pores. It is seen from Figure 1d that the powder was extremely loose, and the pore size varied between 0.5 and 4 μm. This result confirms that glucose was effective in producing fine particles by the formation of carbon, which acted as the dispersing medium of the oxide particles. Figure 1c,d and Figure 1e,f compare the features of the combusted powder with a constant U/Co ratio (1.2) and varied C/Co ratios. The particles observed in Figure 1e,f were visibly larger than those in Figure 1c,d, and few pores were present on the surface of the powder. During the combustion process, the dehydration and decarbonization of glucose consumed excessive heat, resulting in the decrease of combustion temperature [16]. Moreover, excess glucose brought about the postcombustion process, leading to the coarsening of particles.

**Figure 2:**
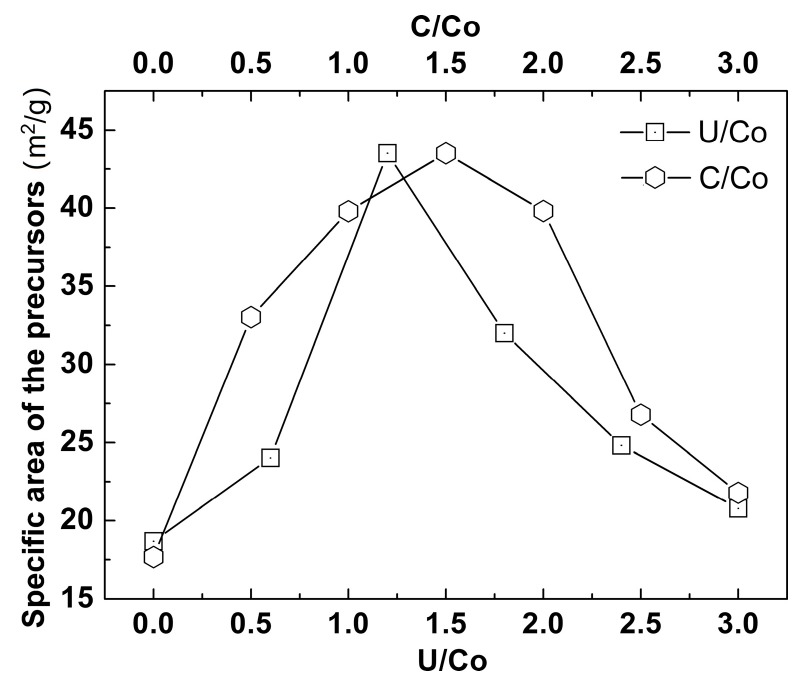
Dependence of specific surface area of the combusted powder on the U/Co and C/Co molar ratios of the solution precursor.

Figure 2 presents the SSA of the combusted powder made from the solution precursor with varied U/Co and C/Co ratios. The two curves shown in Figure 2 demonstrate a similar variation trend. However, it is noted that the SSA was more sensitive to the U/Co ratio than the C/Co ratio. Without the addition of urea, the SSA of the combusted powder was measured to be only 17.7 m^2^/g. As the U/Co ratio increased, a higher specific surface area was achieved, and the highest SSA of the powder reached 43.5 m^2^/g in the case of U/Co = 1.2. However, a further increase of the U/Co ratio induced a decrease in SSA, and the SSA of the combusted powder with U/Co = 3.0 was as low as 20.8 m^2^/g. Therefore, both excessive and insufficient ratios of U/Co and C/Co are unsuitable for the preparation of Co-Ni-W-based alloy powder, resulting in the incomplete combustion of the reactant or the coarsening of the particles due to partial sintering [17,18]. In order to produce powder with small particle sizes and high SSA, it is necessary to adjust the U/Co and C/Co ratios to around 1.2 and 1.5, respectively.

**Figure 3:**
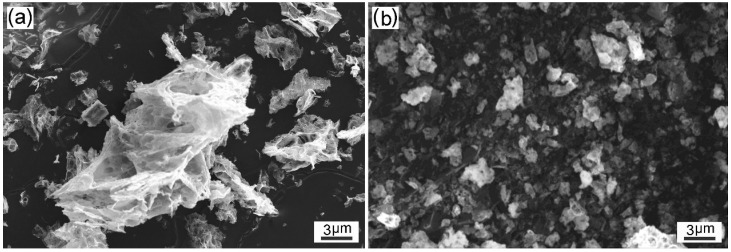
SEM images of the combusted powder synthesized in different media: (**a**) water; (**b**) alcohol.

Figure 3 compares the SEM images of the combusted powder prepared from the solution precursor (U/Co = 0.5, C/Co = 1.5) using the solvent of water and alcohol. The particle size of the powder synthesized in aqueous medium was around 1–21 μm, which is much larger than that of the powder synthesized in alcohol medium (0.4–3 μm). As more gases were liberated from the solution precursor with the use of alcohol, the agglomerated powder was effectively disintegrated into fine particles. The SSA of the powder synthesized in alcohol medium reached 57.6 m^2^/g, which is much higher than that of the powders prepared in water medium (43.5).

**Figure 4:**
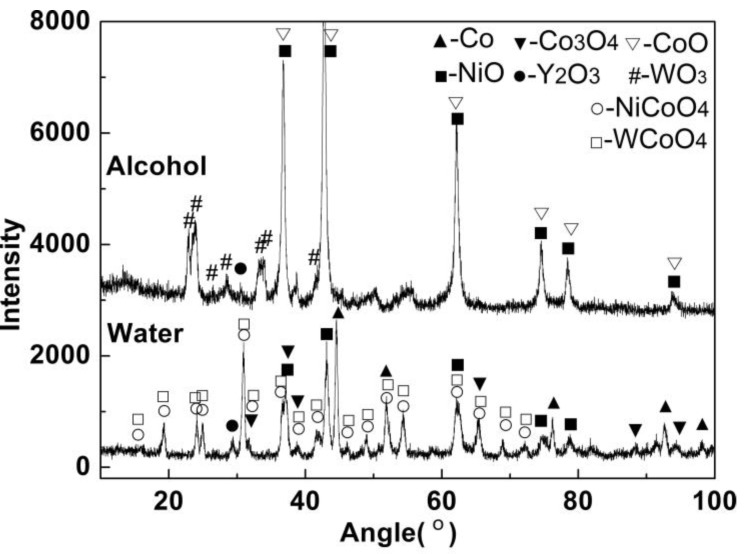
X-ray diffusion (XRD) patterns of the combusted powder prepared in different media.

Figure 4 demonstrates the XRD patterns of the combusted powder prepared in aqueous and alcohol media. The predominant phase in the powder prepared in aqueous medium consisted of Co (PDF Card No.: 15-0806), NiO (PDF Card No.: 44-1159), Y_2_O_3_ (PDF Card No.: 43-0661), NiWO_4_ (PDF Card No.: 15-0755), CoWO_4_ (PDF Card No.: 15-0867), WO_3_ (PDF Card No.: 32-1395), and Co_3_O_4_ (PDF Card No.:42-1467). As for the powder synthesized in alcohol medium, the main constituents were CoO (PDF Card No.: 43-1004), NiO, and WO_3_, and the peaks of NiWO_4_ and CoWO_4_ disappeared. It was an indication that complete combustion was achieved. The remnant of NiWO_4_ and CoWO_4_ revealed the relatively low combustion temperature and the incomplete reaction of tungstate. This is in agreement with the combustion temperature measurement that the combustion temperature of the precursor using alcohol medium is much higher than that of the precursor using aqueous medium. The presence of Co in the powder synthesized in aqueous medium suggests that partial cobalt oxide was reduced, which can be attributed to the reduction action of the dehydrated product (carbon) [13]. Due to the higher combustion temperature, Co was transformed to CoO completely. Additionally, carbon was not detected by XRD analysis, indicating that the carbonic residues were burned out in air atmosphere during the postcombustion process.

### 3.2. Characteristics of the Reduced Powder

**Figure 5:**
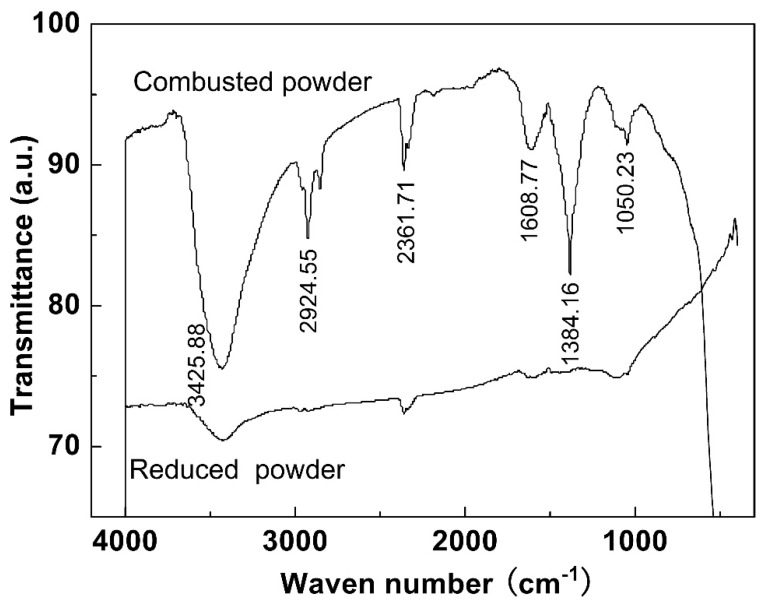
The Fourier transform infrared (FT-IR) spectra of the combusted powder and the powder reduced at 700 °C.

Figure 5 shows the FT-IR spectra of the combusted powder and the reduced powder. Strong transmittance peaks observed at 3425 cm^−1^ in the spectra of combusted powder were assigned to the OH^−^ group of water or moisture present on the surface of the powders. The combusted power also shows some bands due to the organic residues that resulted from the combustion reaction, such as the band at 1608.8 cm^−1^ assigned to C=O stretching vibration, the band at 2924.6 cm^−1^ assigned to C–H stretching vibration, and 1050.2 cm^−1^ which may be due to C–N stretching vibration. Another strong absorption band at 1384 cm^−1^ was assigned to the stretching modes of NO_3_^−^, suggesting the presence of residual nitrate. The absorption band of NO_3_^−^ disappeared in the reduced powder, indicating the complete decomposition of yttrium nitrate and cobalt nitrate during the reduction process at a high temperature (700 °C). The peak at around 485 cm^−1^ in the spectra of the reduced powder was attributed to the metal-oxide (Y–O) mode, gives clear evidence about the presence of crystalline Y_2_O_3_.

**Figure 6:**
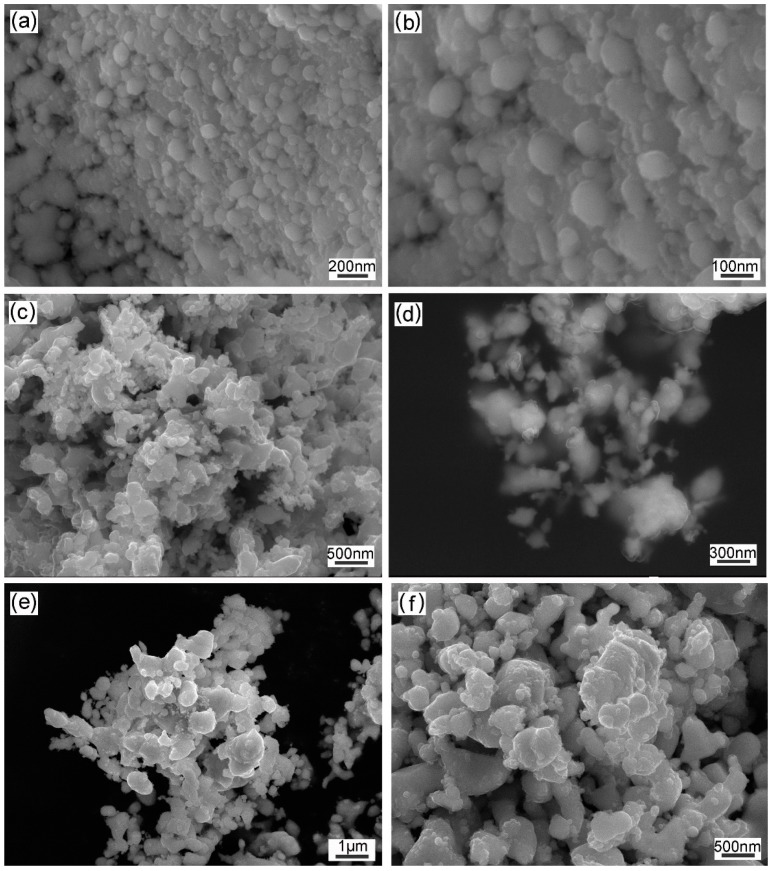
SEM images of the powder reduced at various temperatures of (**a**,**b**) 500 °C; (**c**,**d**) 700 °C; (**e**,**f**) 900 °C.

Figure 6 displays the SEM photographs of the powder reduced at various temperatures in the range of 500–900 °C. As for the powder reduced at 500 °C, lamellar powder was observed at low magnification, as shown in Figure 6a. At high magnification, this lamellar powder consisted of well-distributed spherical oxide particles, and the size of the individual particles were only 40–106 nm, as shown in Figure 6b. The lamellar powder is the soft agglomeration of nanosized particles, which can be separated by grinding. Due to the faster growth of crystallites at higher temperatures, aggregation of the particles was observed at 700 °C, as shown in Figure 6c,d. It is seen in Figure 6e,f that the particle’s surface started to melt at 900 °C, resulting in the formation of imperfect spherical particles and partially fused particles. The average particle size of the Co-Ni-W-based ODS powder grew from 65 to 260 nm in the reduction temperature range of 500–900 °C.

**Figure 7:**
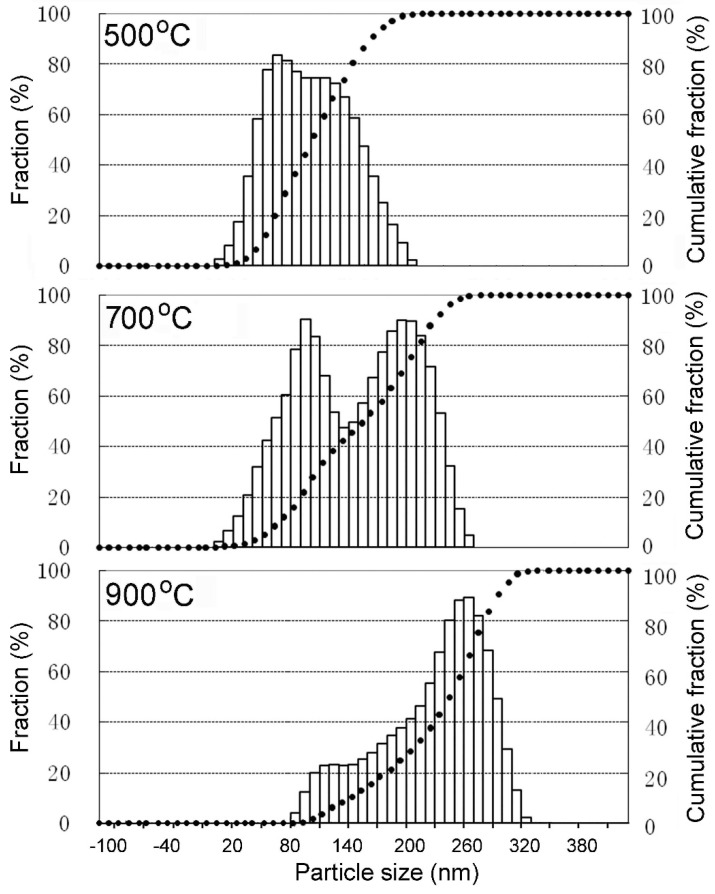
Particle size distribution of the powder that was reduced at the temperature range of 500–900 °C.

The powder was reduced at the temperature range of 500–900 °C, and the particle size was measured by image analysis of the SEM images, as shown in Figure 6. The particle size distribution is given in Figure 7. For the powder reduced at 500 °C, the particle size of is in the range of 10–200 nm. This revealed that the distribution peaks of the coarse particle shift gradually towards a larger value with increasing reduction temperature. These nanosized particles exhibited relatively high coarsening rate, and the average particle sizes of the powder reduced at 500, 700, and 900 °C were 110, 156, and 260 nm, respectively. 

**Figure 8:**
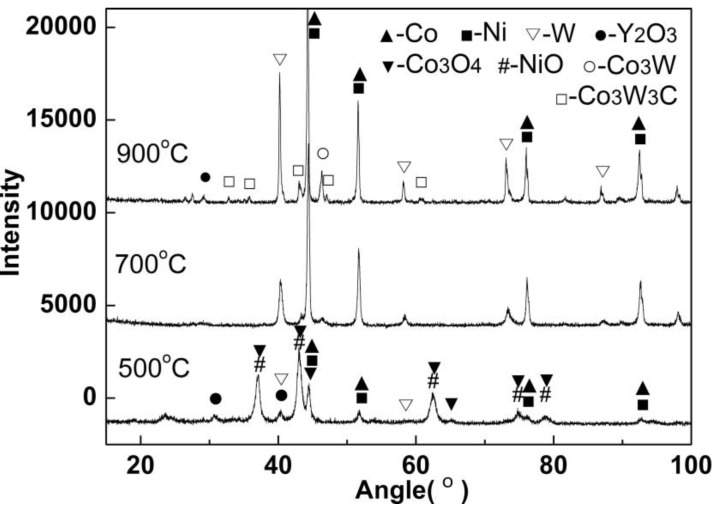
XRD patterns of the powder reduced at the temperature range of 500–900 °C.

Figure 8 shows the XRD patterns obtained for the combustion-synthesized powder and the powder reduced at the temperature range of 500–900 °C. Relatively broad peaks remained for the powder reduced at 500 °C, indicating the small crystalline size of the powder. The sample reduced at 500 °C comprised of Co, Co_3_O_4_, NiO, Y_2_O_3_, and W, indicating that the reduction reaction was not completed at 500 °C. The XRD patterns of the sample reduced at 700 °C show obvious crystallization of the powder. The samples reduced at either 700 and 900 °C were a mixture of Y_2_O_3_ and Co, and the peaks of the 900 °C sample were more intense than those of the 700 °C sample. This implies that the reduction reaction was completed in both the 700 and 900 °C samples. It was noted that the higher temperature of 900 °C induced the formation of a small amount of Co_3_W and Co_3_W_3_C, due to the contamination of carbon residuals.

**Figure 9:**
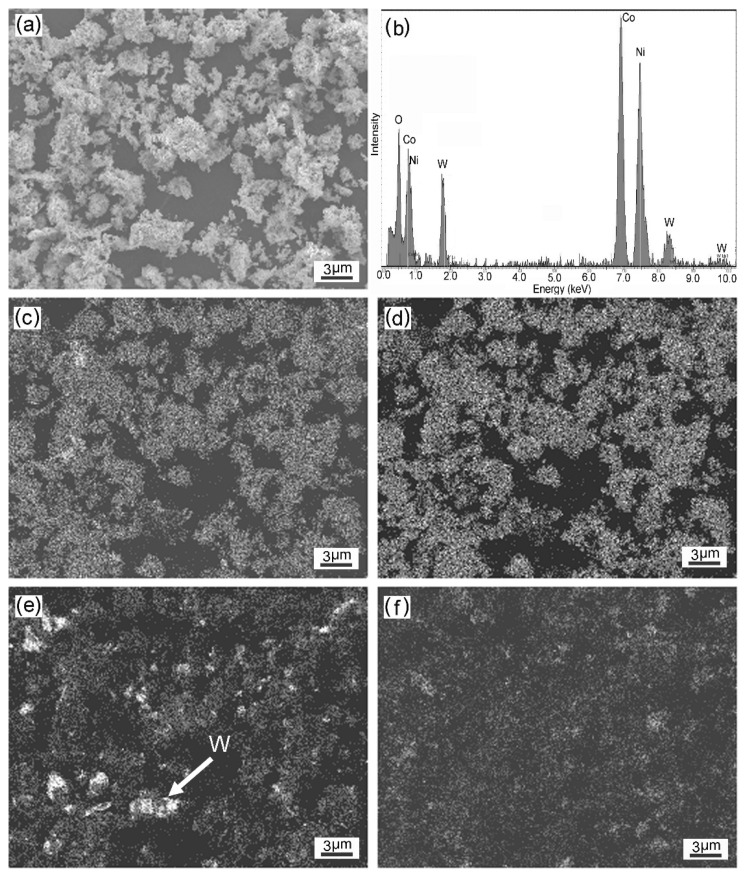
The EDS analysis (**b**) of the resulting Co-Ni-W-based ODS alloy powder (**a**) and corresponding map distribution of the alloying elements: (**c**) Co; (**d**) Ni; (**e**) W; (**f**) Y.

Figure 9 shows the energy dispersive spectroscopy (EDS, INCA, Oxford Instruments, Oxford, UK) analysis of the resulting Co-Ni-W-based ODS alloy powder. It revealed that the powder consisted mainly of Co, Ni, W, and the composition of the powder mixture was 49.8 wt. % Co-29.3 wt. % Ni-19.4 wt. % W-1.2 wt. % Y. Uniform distribution of Co, Ni, and Y was observed. Some relatively large W-rich zones were present (marked by W), which may be attributed to the high density of W. Further improvement in the degree of homogeneity of W particles is needed.

## 4. Conclusions

Co-Ni-W-based ODS alloy powder was successfully synthesized by low-temperature combustion synthesis. The combusted powder and the reduced powder were characterized, and the following conclusions could be drawn:

(1) Co-Ni-W-based ODS alloy powder exhibits nanosized particle size, high specific area, and a relatively homogeneous distribution of alloying elements. The SSA of 43.5 m^2^/g was obtained under the optimized conditions of U/Co = 1.2 and C/Co = 1.5.
(2) The lamellar Co-Ni-W-based ODS alloy powder with sizes of 1–21 μm was the soft agglomeration of a population of nanosized (65 nm) particles.
(3) The powder synthesized in alcohol medium exhibited much smaller particle size and larger SSA than that of the powder synthesized in aqueous medium
(4) Nanoparticles grew from 65 to 260 nm in the reduction temperature range of 500–900 °C.
